# Development of a Low-Cost Microphotoreactor from Recycled Materials: Application to Nb_2_O_5_@H_2_TPP-Catalyzed Methylene Blue Degradation

**DOI:** 10.1021/acsomega.5c08779

**Published:** 2026-01-22

**Authors:** Lívia Silva de Andrade, João Victor Docílio Pereira, Tiago Souza Brasil, Clarissa B. da. S. Neves, Felipe Breno Campos Marinho, Júlio Santos Rebouças, Sivanildo da Silva Borges, Fábio Santos de Oliveira, Clarivaldo Santos Souza, Gilson DeFreitas-Silva, Denilson Santos Costa, Vinicius Santos da Silva

**Affiliations:** † Centro de Ciências Exatas e Tecnológicas, 186074 Universidade Federal do Recôncavo da Bahia, 44380-000 Cruz das Almas, Bahia, Brazil; ‡ Instituto de Química, 28111 Universidade Federal da Bahia, 40170-115 Salvador, Bahia, Brazil; § Departamento de Química, Centro de Ciências Exatas e da Natureza, Paraiba Federal University, 58051-900 João Pessoa, Paraíba, Brazil; ∥ Centro de Ciências da Saúde, Universidade Federal do Recôncavo da Bahia, 44570-000 Santo Antônio de Jesus, Bahia, Brazil; ⊥ Departamento de Química, Instituto de Ciências Exatas, 133635 Universidade Federal de Minas Gerais, 31270-901 Belo Horizonte, Minas Gerais, Brazil

## Abstract

A low-cost, robust, and easy-to-operate photoreactor with automatic temperature control, achieved through heat sinks, cooling fans, a temperature sensor, and a microcontroller (Arduino Nano), was manually constructed using predominantly discarded materials, without the need for sophisticated instrumentation. The light source employed was a 3 W RGB LED lamp with infrared (IR) remote control. The electromagnetic radiation spectra (white, blue, green, and red) were determined by UV–vis spectroscopy; additionally, the irradiance and luminous flux of these radiations were evaluated. For luminous flux determination, a lux meter was developed based on a BH1750-FVI sensor coupled to the Arduino Nano. The characterizations indicated that red radiation exhibits the highest irradiance and luminous flux values when compared to blue and green radiations. The estimated cost for constructing the photoreactor was US$ 41.50, which is significantly lower than that of commercially available photoreactors, whose prices typically exceed US$ 3000.00. To validate the performance of the photoreactor, the photocatalytic degradation of methylene blue (MB) was carried out using a novel Nb_2_O_5_@H_2_TPP material as the photocatalyst. This material was synthesized by physical mixing of niobium pentoxide (Nb_2_O_5_) and 5,10,15,20-tetraphenylporphyrin (H_2_TPP). The resulting photocatalyst was comprehensively characterized by X-ray diffraction, UV–vis diffuse reflectance spectroscopy (UV–vis/DRS), scanning electron microscopy, zeta potential measurements, infrared spectroscopy, and thermogravimetric analysis. The MB degradation reactions were initially conducted following a factorial experimental design. This analysis identified MB concentration as a negative factor in the degradation percentage, catalyst mass as a positive factor, and reaction time as a nonsignificant variable within the time range explored. Based on these findings, MB degradation reactions were performed under different ranges of incident light wavelengths (white, blue, green, and red). The MB degradation percentages observed were 32% under white light, 34% under blue light, 42% under green light, and 44% under red light exposure.

## Graphical abstract

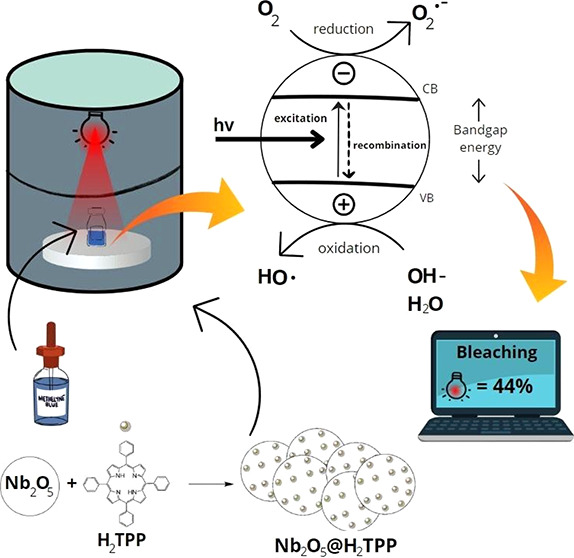

## 1 Introduction

The contamination of water bodies with dyes, mainly from the textile industry, is one of the main sources of environmental pollution. These include methylene blue (MB),^1,2^ Figure 1a, which is a water-soluble substance belonging to the phenothiazine class.^2,3^ The use of this dye in the textile industry is common in the processing of polyesters and nylons, as well as in medicine, in cancer tests. The discharges generated, mainly from industries, are harmful to aquatic biota, even at low concentrations. This occurs because methylene blue exhibits high molar absorptivity, which reduces the transparency of the aqueous medium and hinders light penetration into deeper regions of rivers and lakes. This limitation negatively affects photosynthetic activity, consequently decreasing the levels of dissolved oxygen in the aquatic ecosystem.^2,3^

**Figure 1:**
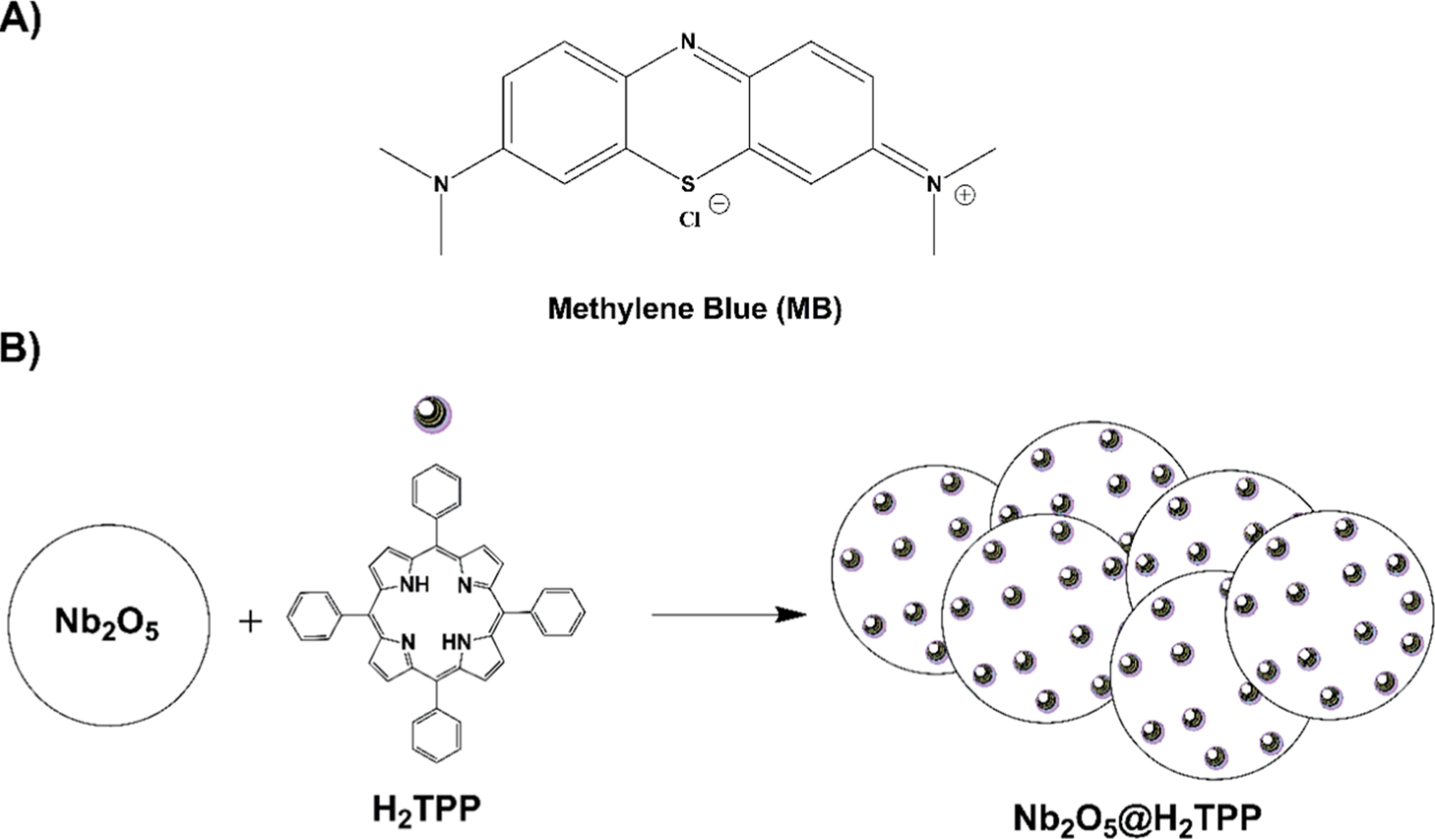
(A) Chemical structure of methylene blue; (B) structural representation of the photocatalytic material prepared.

Conventional methods used for the removal and/or degradation of MB include filtration, adsorption/biosorption,^4,5^ flocculation^6^ and biodegradation;^7^ these treatment methods have advantages and limitations in terms of cost, feasibility, effectiveness and impact on the environment.^8^

The literature describes alternative strategies for removing and/or degrading this pollutant, such as biomimetic catalysis^9,10^ and heterogeneous photocatalysis.^11–15^ The latter is based on the use of radiation in the Ultraviolet–visible (UV–vis) region to generate, in the presence of a photocatalyst, free radicals that act in the partial or total transformation of pollutants into substances that are less harmful to the environment.^3,16,17^ The characteristics of the light source and the photocatalyst are therefore essential for an effective process.

However, the experimental application of heterogeneous photocatalysis is often limited by the high cost of commercial photoreactors, which restricts access to this technique for many research groups and academic laboratories. Given the importance of electromagnetic radiation, it is crucial to use a photoreactor with specific structural characteristics, such as a shape that favors a greater incidence of radiation;^18^ the photoreactor should also be internally coated with a reflective material, be amenable to selecting the light source of different wavelengths and power, and be built with a temperature control system.^19^ This last aspect is particularly important in open systems, where prolonged irradiation can lead to solvent evaporation and thermal fluctuations that can significantly affect photocatalytic performance.

Different types of laboratory-scale photoreactors are described in the literature.^20^ The shape generally used is an annular one, as it increases reflection in the center, where the reactions will take place. As a light source, Light Emitting Diodes (LEDs)^18^ or mercury lamps of different powers and wavelengths are used. LEDs are more advantageous because they use a specific wavelength range, which increases the efficiency of the incident radiation, and there is no need to use filters.^21^ It is worth mentioning that most of the work carried out does not include an electronic system for temperature control, which is essential, since exposure to light for a prolonged period can lead to heating of the reaction system and directly influence the results.^20^ Although most of the microphotoreactors described in the literature are robust and provide high yields in pollutant degradation reactions, their widespread use is often limited due to their high cost of production.

While TiO_2_ and ZnO remain the most widely employed materials in heterogeneous photocatalysis owing to their high stability and low cost,^17,22–24^ these conventional oxides present challenges related to charge recombination kinetics and band alignment specificity. In this context, Nb_2_O_5_ has recently attracted significant attention as a highly promising alternative,^25^ offering intrinsic physicochemical properties—such as superior chemical stability and a more negative conduction band (CB) potential—that are essential for enhancing efficiency and selectivity in solar–driven reactions.^26^ Furthermore, the presence of unique Lewis and Brønsted acid sites on the Nb_2_O_5_ surface contributes significantly to its catalytic versatility and selectivity.^27,28^ Although its wide band gap 2.6 to 4.1 eV (depending on the structure)^26^ restricts its activation to UV radiation, thus limiting efficiency under solar light, recent research efforts are focused on mitigating this drawback through electronic and structural engineering and which has been applied in different areas.^29^ Furthermore, considering Brazil’s status as the holder of the world’s largest niobium reserves (∼98%),^30^ the development of niobium-based technologies transcends mere material choice, reinforcing a strategic national interest in fostering innovative and sustainable environmental applications.^30^

One of the limitations of using semiconductors such as TiO_2_, ZnO and Nb_2_O_5_ as photocatalysts is their low efficiency in absorbing visible radiation, as they are preferentially activated by UV light.^31^ In this context, different strategies have been developed by the scientific community, such as coupling semiconductors to dyes, which can act as sensitizers that are activated by light in the visible region.^12,16,32–35^ Thus, porphyrins, which are aromatic and highly conjugated compounds, stand out, as they exhibit intense absorption in the visible region due to an extensive π-electron conjugation system.^36,37^ Various studies have used porphyrins associated with semiconductor oxides,^32,33,37–39^ but there are no reports of studies using Nb_2_O_5_ associated with porphyrins as photocatalysts for pollutant photodegradation reactions under visible light.

In this context, the main objective of this work is to develop and characterize a low-cost, easily reproducible photoreactor built primarily from recycled materials, equipped with automated temperature control and adaptable radiation sources. To validate its performance, we employed niobium pentoxide associated with 5,10,15,20-tetraphenylporphyrin (H_2_TPP) as a photocatalyst for the visible-light degradation of methylene blue—an unprecedented system in the literature, Figure 1b.

## 2 Results and Discussion

### 2.1 Photoreactor

In constructing the photoreactor, cylindrical Medium-Density Fiberboard niches were chosen, resulting in a ring-shaped arrangement. This geometry enhances the propagation and reflection of radiation toward the center.^20^ Internally, the cylinders were lined with aluminum foil—a low-cost, corrosion-resistant, flexible, and highly reflective material—with the purpose of reducing radiation absorption and increasing reflection.^40^

The photocatalytic reactions are carried out in penicillin flasks fixed to a sample holder molded with hot glue, coated with aluminum foil, and positioned at the central part of the chamber (Figure 2A). The lamp is attached to the other chamber (upper part of the photoreactor). The chambers are not fixed to each other, which facilitates their movement and adjustment. The operation of the photoreactor is straightforward: once the temperature parameters are set, it only needs to be connected to the power supply. Then, the desired light color for the reaction is selected, and the reaction flasks are placed in the sample holder.

**Figure 2:**
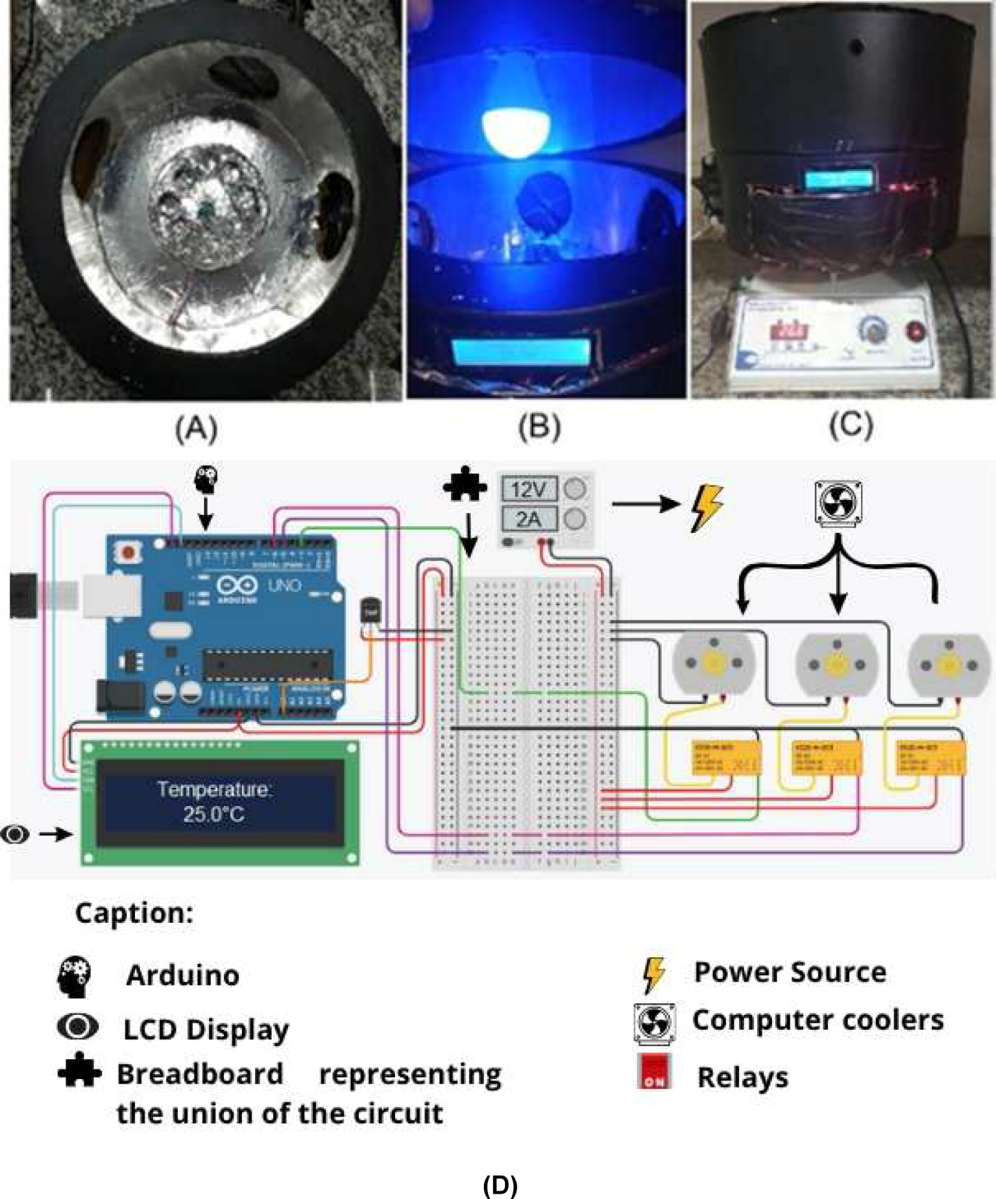
Photographic images of the photoreactor. (A) Top view; (B) front view with the photoreactor open; (C) front view with the photoreactor closed (D) schematic diagram of the photoreactor electrical circuit.

Regarding temperature control, three coolers were arranged: two positioned opposite each other and a third placed between them. All coolers were configured to exhaust heat, removing it from the internal environment (Figure 2A). An Arduino Nano was used as the microcontroller; although primarily a prototyping platform, it was selected for its low cost and ease of use.

Temperature readings were obtained using a DHT11 sensor, capable of measuring temperatures ranging from 5 to 50 °C. The output is displayed on an LCD with an I2C module (Figure 2C); both components were chosen for their affordability and simplicity. The I2C module was employed to reduce the number of wires and connections, enabling the use of the Arduino Nano instead of Arduino Mega or Uno. Similarly, a PCB board was used to unify the power supply for the Arduino and the external source, minimizing excess wiring and connections.

The code was written in C++ using the Arduino IDE (Supporting Information). To maintain a constant temperature of (25 ± 2) °C, the Arduino was programmed to activate the three coolers when the temperature exceeds 25 °C, using a 12 V relay (illustrated in Figure 2D). The relay shown is illustrative only, as its electronic characteristics differ; the actual relay used has the reference code JQC3F-05VDC-C. When the temperature falls to 24 °C or below, the system is programmed to turn off the coolers using the same relay (Figure 2D). The light source used was an RGB LED lamp with infrared (IR) remote control. LED lamps offer advantages over mercury lamps, such as the ability to select specific wavelength ranges, ease of implementation, and minimal heat generation.

The emission spectra of the LED lamp for each selected color (blue, green, and red) and for white light—which consists of the combined, simultaneous emission of blue, green, and red LEDs—are shown in Figure 3.

**Figure 3:**
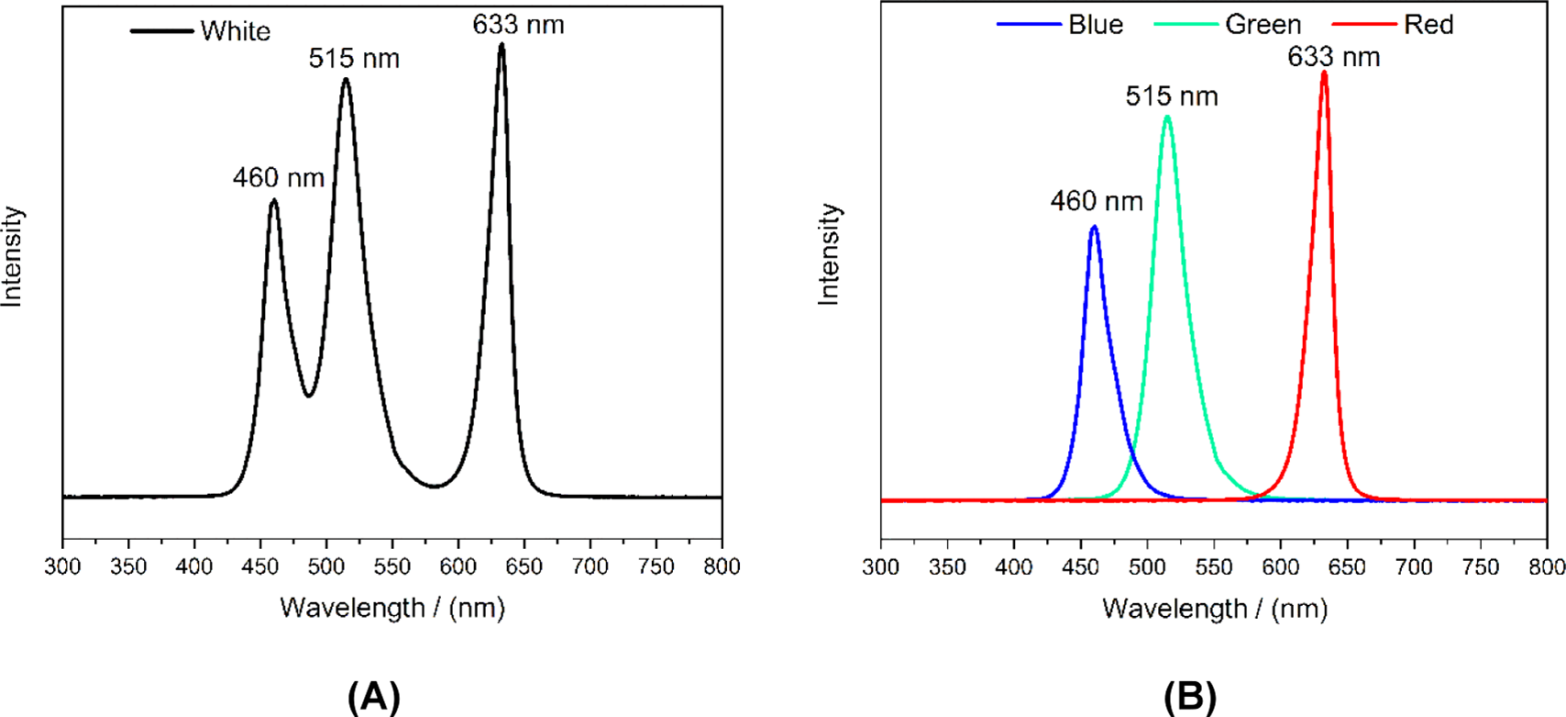
Emission spectra of radiation in (a) white light and (b) in blue, green, and red colors from the RGB LED lamp.

Regarding the emission spectra, each selected color exhibits a relatively broad emission range, with green showing the widest range (∼475 to 575 nm) and red the narrowest (∼600 to 660 nm).

When calculating the illuminance for each color analyzed in this study (Table 1), it was observed that white light exhibited the highest illuminance and luminous flux, as expected, since it results from the combined emission of the red, green, and blue LEDs. This was followed by green, red, and blue light, with blue having the lowest lumens.

**Table 1:** Relationship between Illuminance, Luminous Flux, and Colors

colors	illuminance (l×)	luminous flux (lumens)
blue	72.00 ± 0.46	29.38 ± 0.25
green	439.72 ± 1.15	179.40 ± 0.91
red	251.10 ± 1.85	102.44 ± 0.55
white	705.06 ± 0.69	287.66 ± 1.45

A detailed understanding of the irradiation characteristics is crucial, as it provides the foundation for ensuring reliable comparison and reproducibility of photochemical reactions. In this context, the irradiance of the RGB LED lamp for white, blue, green, and red light was measured using a fiber-optic spectrometer (Figure 4). The highest normalized absolute irradiance values were observed for the white and red-light emissions.

**Figure 4:**
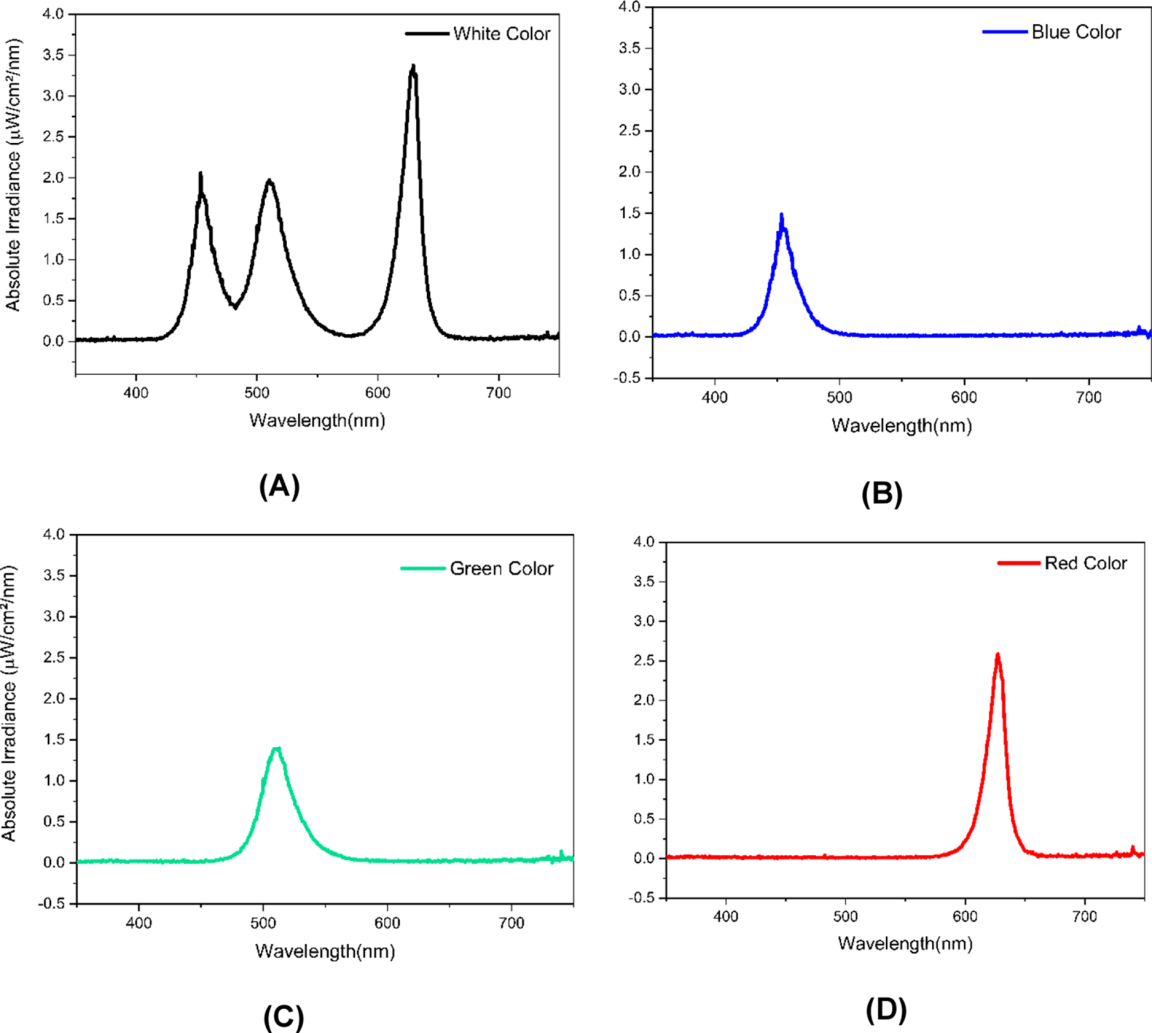
Normalized absolute irradiance spectra of the RGB LED lamp in the 350–750 nm range for (a) white, (b) blue, (c) green, and (d) red light.

The photoreactor was mainly assembled using repurposed materials; however, a few electrical components were acquired, as detailed and costed in the Supporting Information (Table S1). The total assembly cost was US$ 41.50, confirming the high economic feasibility of the proposed design. In comparison with the low-cost microphotoreactor developed in this study, commercially available photoreactors remain substantially more expensive. As summarized in Table S3, the price of commercial systems currently ranges from approximately US$ 3240.00 for compact LED-based platforms, such as the SynLED Parallel Photoreactor 2.0 (Sigma-Aldrich), to values above US$ 8900.00 for more advanced models like the Penn PhD Photoreactor M2 (Sigma-Aldrich). Even higher-cost configurations are found among specialized manufacturers, including the PhotoRedOx Box UV–vis (HepatoChem; US$ 5250.00) and the LZC series photoreactors from Luzchem Research, which can exceed US$ 15,900.00 depending on the configuration. This broad price range underscores the economic barrier associated with acquiring commercial photoreactors and highlights the relevance of developing accessible, low-cost, and modular alternatives for photochemical research. Despite the functional and structural differences between the systems, the substantially lower cost and the ease of assembly of our prototype highlight its potential as a truly accessible and cost-effective alternative for laboratory-scale photochemical applications.

### 2.2 Photocatalytic Material

The procedure adopted to obtain Nb_2_O_5_ from the calcination of ammoniacal niobium oxalate (NH_4_[NbO­(C_2_O_4_)_2_(H_2_O)]­3­(H_2_O)) was relatively simple and inexpensive.^41^ This material was characterized by X-ray diffraction (XRD), surface area and pore volume distribution, scanning electron microscopy (SEM) and Ultraviolet–Visible Spectroscopy (UV–vis) by diffuse reflectance.

XRD analysis was carried out at room temperature (25 °C) in order to identify the phase(s) present in the sample synthesized from the calcination of ammoniacal niobium oxalate. Comparison of the diffractogram of the product (Figure 5) with patterns from the ICDD PDF2 Release 2004 database revealed that the sample contained only the Nb_2_O_5_ phase with a hexagonal crystal structure (PDF# 00-007-0061), with peaks assigned accordingly. The XRD data provided evidence that the procedure used was effective in obtaining the material of interest and no segregation of secondary phases were verified. The incorporation of the porphyrin into niobium pentoxide did not alter the crystalline structure of Nb_2_O_5_ (Figure S2, Supporting Information).

**Figure 5:**
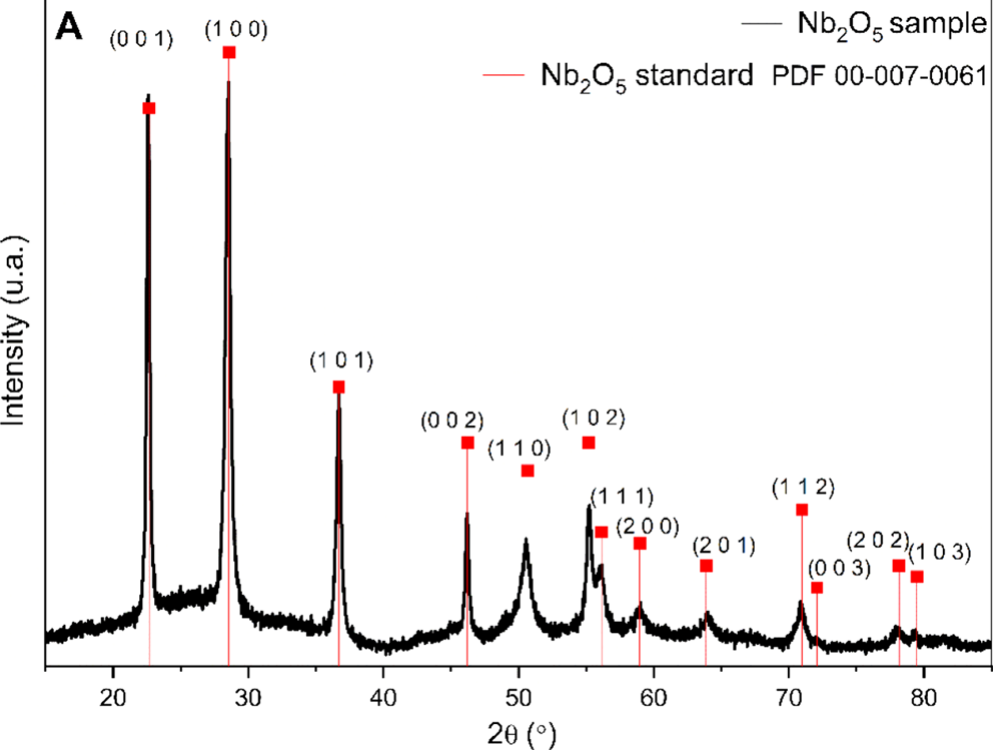
Diffractogram of Nb_2_O_5_.

Using the Brunauer–Emmett–Teller (BET) method, a value of 19.7 m^2^ g^–1^ was obtained for the specific surface area, using Barrett−Joyner−Halenda (BJH) method, a value of 31.3 nm was obtained for the desorption average pore size of Nb_2_O_5_ (Figure S3, Supporting Information) and N_2_ adsorption–desorption isotherm type III of IUPAC classification (Figure S4, Supporting Information), indicating weak interaction between absorbent and absorbed, which belongs to macroporous material or mesoporous material with irregular porosity material.^42,43^ In general, Nb_2_O_5_ present only external porosity (i.e., meso- and macro-pores) without measurable microporosity. Reagents and synthesis methods influence the characteristics of the material obtained. It has been found that heat treatments of Nb_2_O_5_·H_2_O result in a deterioration of its textural properties, with a substantial decrease in specific surface area and pore volume. Treatment in air at a temperature of 550 °C (conditions used in this work) resulted in a drop of over 90% in *S*_BET_ as compared to *S*_BET_ of Nb_2_O_5_·*n*H_2_O.^44^

The association of H_2_TPP with Nb_2_O_5_ was achieved through a physical mixture, in which the porphyrin, previously dissolved in dichloromethane, was added to the oxide. The resulting suspension was kept under mild heating and magnetic stirring until complete solvent evaporation. This procedure favored the formation of a visually uniform material with a brownish coloration, suggesting a good dispersion of the porphyrin on the oxide surface. In order to characterize the material after association of H_2_TPP in Nb_2_O_5_, Infrared Spectroscopy Analysis with Fourier Transform (FTIR) (Figure 6a), thermogravimetric analysis (TGA) (Figure 6b) and Scanning Electron Microscopy (SEM) (Figure 7a,b) were performed. However, since the porphyrin content in the material is below 1 wt %, techniques such as TGA, FTIR and SEM do not exhibit sufficient sensitivity to unequivocally detect its presence.

**Figure 6:**
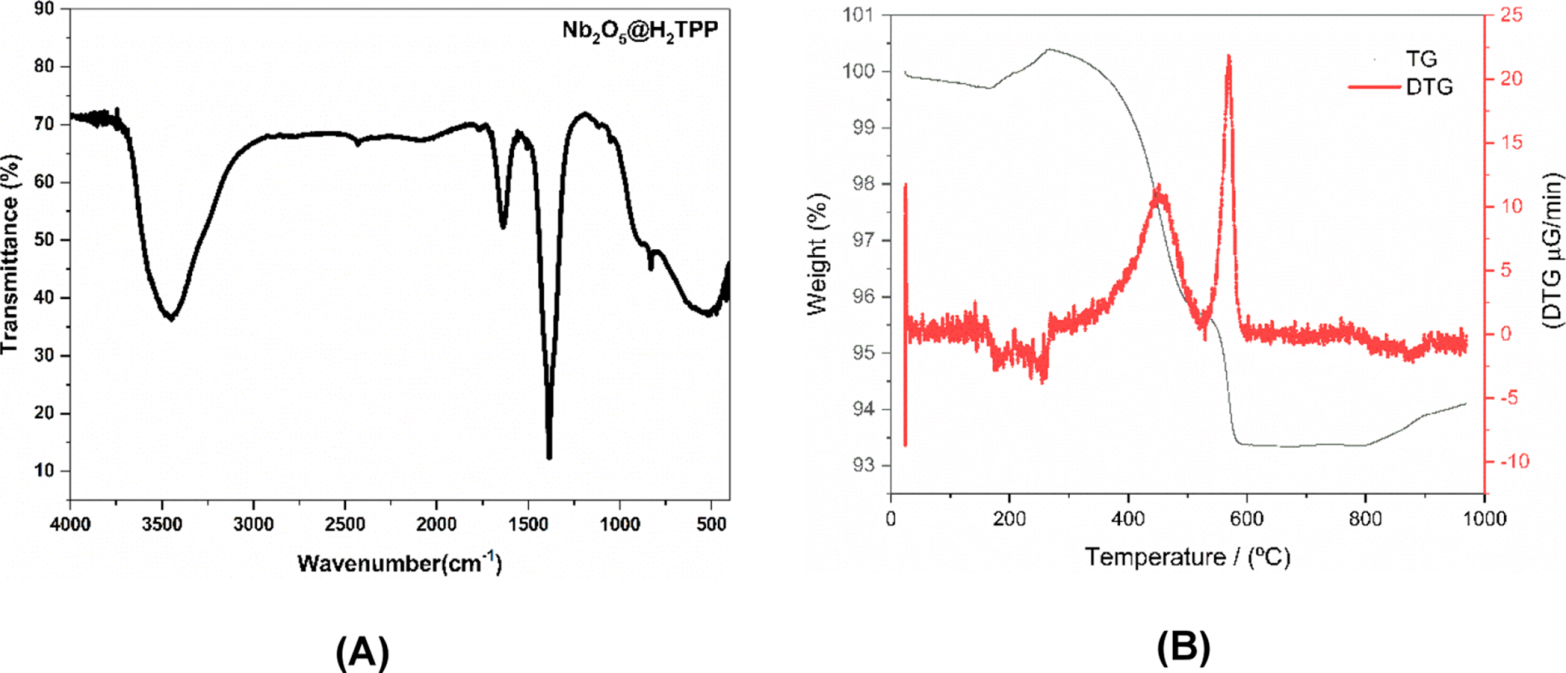
(A) Infrared absorption spectra of Nb_2_O_5_@H_2_TPP in KBr pellets. (B) Thermogravimetric analysis (TGA) curves in black line and derivative thermogravimetry (DTG) curves in red line of Nb_2_O_5_@H_2_TPP.

**Figure 7:**
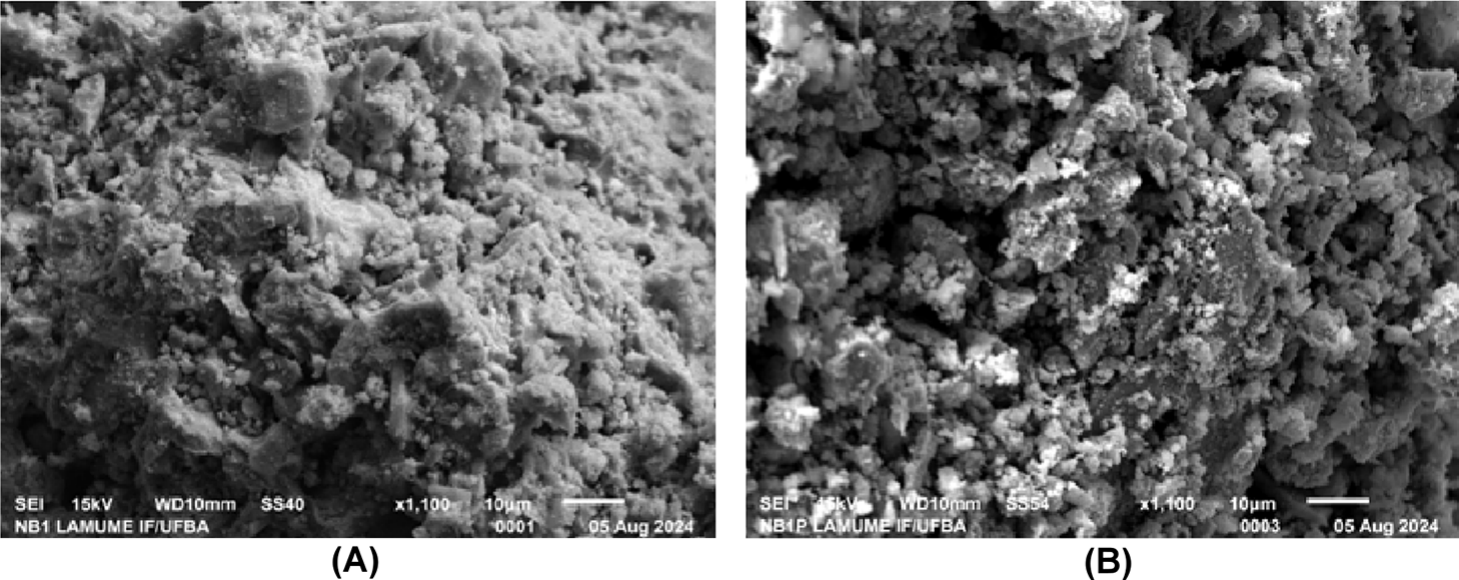
Micrographs corresponding to the materials (a) Nb_2_O_5_ and (b) Nb_2_O_5_@H_2_TPP.

In Figure 6A, a band at 3449 cm^–1^ is assigned to the O–H stretching of Nb–OH and band at 1629 cm^–1^ can be attributed to vibration in water molecules adsorbed on the surface of Nb_2_O_5_. A band at 829 cm^–1^ can be attributed to the symmetric stretching mode of Nb–O surface species and a board band between 764 cm^–1^ and 412 cm^–1^ is associated with the vibrations of Nb–O–Nb bridges in Nb_2_O_5_. Furthermore, band at 1402 cm^–1^ suggested the existence of CO_*x*_.^30,45,46^

When analyzing the thermogram in Figure 6B, a total mass loss of approximately 23% of the initial material can be observed. Six mass loss events can be identified. Those observed at 84 and 142 °C can be attributed to the loss of water molecules adsorbed on Nb_2_O_5_. The third event (253 °C) can be attributed to the elimination of hydration water, while the events observed at 584 °C, 618 °C, and 655 °C can be associated with changes in the crystal structure of Nb_2_O_5_, from T-Nb_2_O_5_ to M-Nb_2_O_5_.^46^

By analyzing the surface morphology of Nb_2_O_5_ and Nb_2_O_5_@H_2_TPP materials using SEM (Figure 7a,b). It is possible to propose that, on the surface of both Nb_2_O_5_ and Nb_2_O_5_@H_2_TPP, the distribution of particle aggregates is not homogeneous in terms of shape and that the association of porphyrin did not significantly alter the surface morphology of Nb_2_O_5_, which is understandable given the relatively small amount of H_2_TPP mixed with Nb_2_O_5_.

The UV–vis/DR spectrum (Figure 8a) for niobium pentoxide shows two bands in the ultraviolet region (maxima at 202 and 294 nm) and a low-intensity band with a maximum at 419 nm, which suggests the low absorption efficiency of this compound in the visible region.

**Figure 8:**
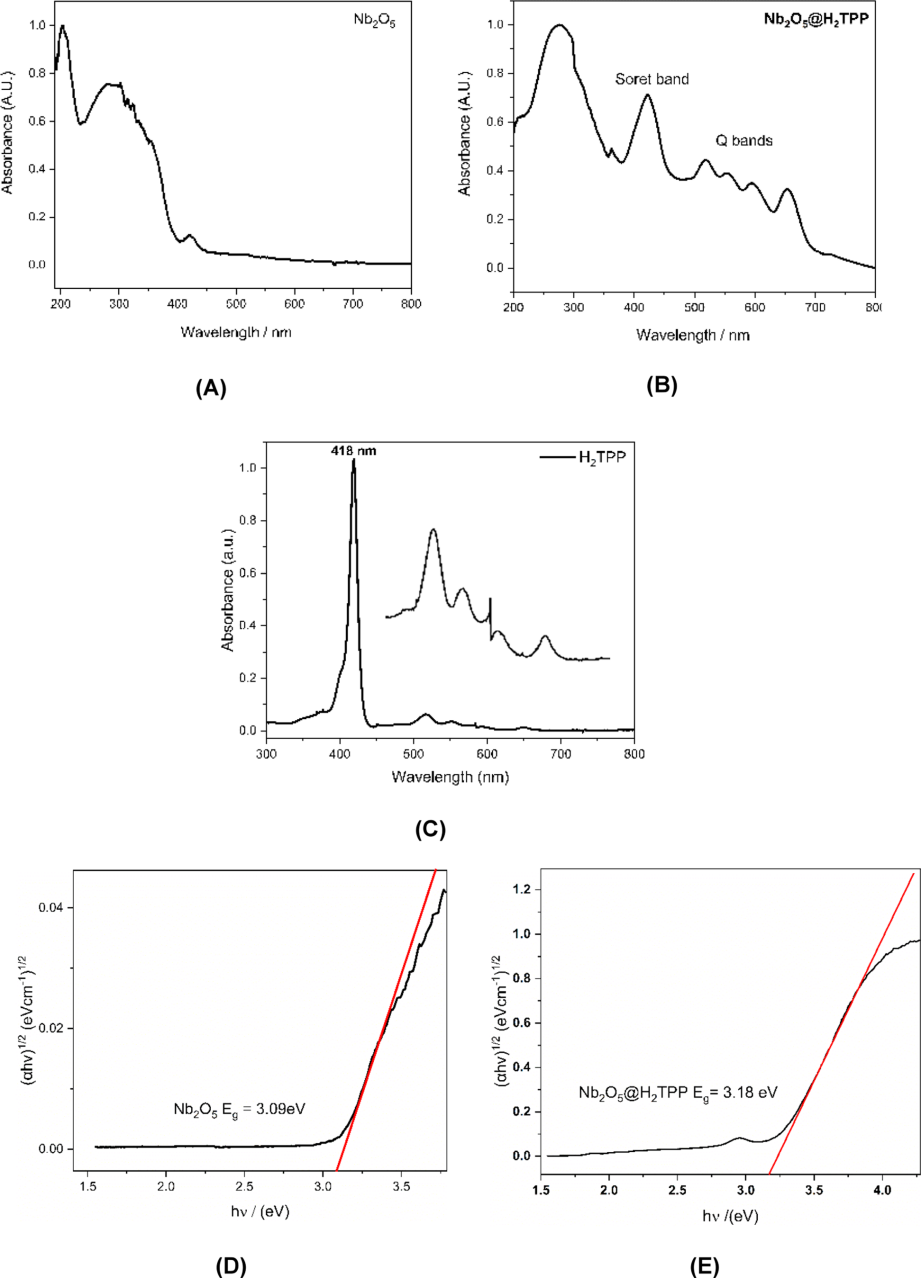
UV/vis-DRS spectrum: (A) Nb_2_O_5_ and (B) Nb_2_O_5_@H_2_TPP. (C) UV/vis spectrum for H_2_TPP in CH_2_Cl_2_. Kubelka–Munk Plot for band gap calculation: (D) Nb_2_O_5_ and (E) Nb_2_O_5_@H_2_TPP.

For the photocatalytic material Nb_2_O_5_@H_2_TPP, the UV–vis/DRS absorption spectrum (Figure 8b) showed the presence of a band at 274 nm (ultraviolet region) associated with Nb_2_O_5_, four low intensity bands between 500 and 700 nm (which are assigned to the porphyrin Q bands) and a higher intensity band at 421 nm (the characteristic porphyrin Soret band) Figure 8c; this characterizes, thus, the successful immobilization of H_2_TPP onto the niobium pentoxide surface. The Q and Soret bands can be associated with the electronic transitions between the two highest occupied π orbitals and the two lowest unoccupied π* orbitals of H_2_TPP, according to Gouterman’s Four Orbital Model.^47,48^

The band gap energy was estimated using the Tauc plot method^49,50^ from the optical absorption data near the band edge, according to eq 1

1 $$ahv={A\left(hv-E_{g}\right)}^{n/2}$$

where α, ν, *A*, and *E*_g_ are the absorption coefficient, light frequency, proportionality constant, and band gap, respectively. In the equation, *n* depends upon the characteristics of the transition in a semiconductor (*n* = 1 for a directly allowed transition and *n* = 4 for indirectly allowed transition, as in this work). *E*_g_ was estimated from the graph of (α*h*ν)^2/*n*^ as a function of h*v*. The *E*_g_ value was determined by extrapolating the linear region of the curve to the *hv* axis, as shown in Figure 8d,e. Using the intersection of the line tangent to the inflection point of the curve (α*hv*)^2/*n*^ versus *hv*, band gap energy values were estimated for Nb_2_O_5_ and Nb_2_O_5_@H_2_TPP at 3.09 and 3.18 eV, respectively (Figure 8d,e), which is in line with the review work described by Pang et al. (2023),^51^ which reports that Nb_2_O_5_ exhibits varying bandgap energies (*E*_g_) ranging from 3.1 eV (semiconducting behavior) to 5.3 eV (insulating behavior).^51^ As the estimated band gap values are very close, it is inferred that the association of H_2_TPP with Nb_2_O_5_ occurred by means of physisorption, with no significant impact on the electronic structure of the semiconductor, which is in line with the work described by Makula, Pacia and Macyk (2018) who demonstrated that when a dye is adsorbed to a semiconductor material it practically does not alter the estimated band gap value.^50^

The conduction band (CB) and valence band (VB) edge positions of Nb_2_O_5_ were estimated according to the empirical approach proposed by Xu and Schoonen (2000),^52^ which correlates the compound’s absolute electronegativity (χ) and its experimental band gap energy (*E*_g_). The absolute electronegativity value adopted for Nb_2_O_5_ was χ = 6.29 eV, as reported in the literature.^52^ The band edge positions (*E*_CB_ for conduction band and *E*_VB_ for valence band) were calculated using the following relationships (eqs 2 and 3)­

2 $$E_{CB}=\chi -E_{e}-0.5E_{g}$$

3 $$E_{VB}=E_{CB}+E_{g}$$

where *E*_e_ = 4.5 eV represents the energy of a free electron on the vacuum scale.

Based on the experimentally determined band gap of *E*_g_ = 3.09 eV, the calculated conduction band *E*_CB_ and valence band *E*_VB_ potentials for Nb_2_O_5_ were 0.24 and 3.33 eV (vs NHE), respectively. These results are consistent with reported literature data and demonstrate that Nb_2_O_5_ possesses a conduction band potential close to the hydrogen reduction level (H^+^/H_2_) and a deep valence band, both characteristics being highly favorable for heterogeneous photocatalytic processes under UV irradiation. The formation of the material by associating H_2_TPP with Nb_2_O_5_ resulted in only a marginal alteration of these potential values. The observed band gap for the Nb_2_O_5_@H_2_TPP material was *E*_g_ = 3.18 eV, with calculated potentials of *E*_CB_ = 0.20 eV and *E*_VB_ = 3.38 eV (vs NHE).

Considering that heterogeneous photocatalytic reactions take place on the catalyst surface, it is essential to understand the surface charge characteristics, as potential environmental pollutants exhibit different electronic properties—existing as cationic, anionic, or neutral species. The zeta potential analysis of Nb_2_O_5_@H_2_TPP at different pH values shows that the material exhibits a positive potential only under strongly acidic conditions (pH 2) Figure 9. Under all other conditions, the observed values are negative, reaching approximately −53 mV at pH 12.

**Figure 9:**
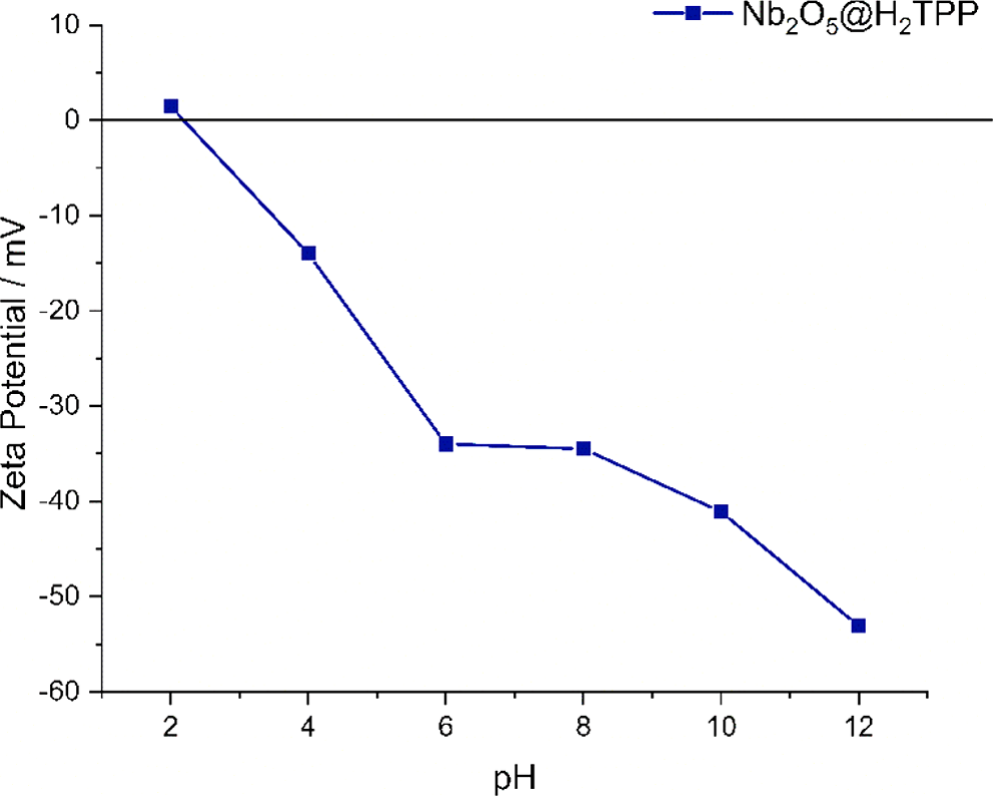
Variation of zeta potential with pH (2–12) for Nb_2_O_5_@H_2_TPP.

### 2.3 Adsorption Assays

The assays to determine the time required to reach adsorption/desorption equilibrium of methylene blue on the photocatalytic materials Nb_2_O_5_ and Nb_2_O_5_@H_2_TPP were conducted under conditions analogous to those used in the photocatalysis reactions, but in the absence of light. The analyses were performed by UV–vis spectroscopy at 30 min intervals (Figure 10).

**Figure 10:**
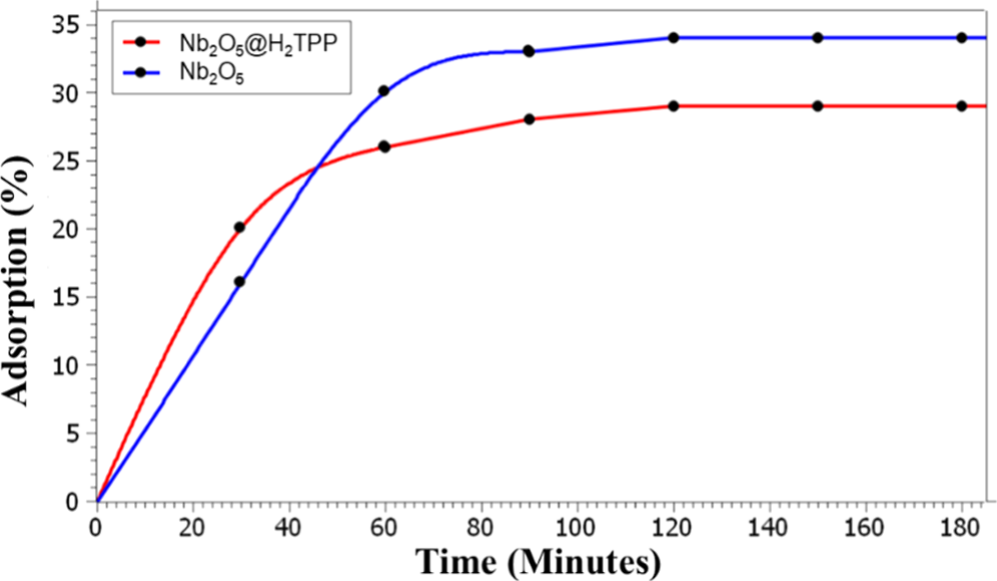
Percentage of methylene blue adsorption as a function of time for 10 mg Nb_2_O_5_ and 10 mg Nb_2_O_5_@H_2_TPP. [MB] = 1 × 10^–3^ mol L^–1^ and 10 mL.

For both materials studied, an increase in adsorption was observed up to 120 min. After this period, the adsorption percentage remained stable until 180 min, indicating that the system had reached adsorption/desorption equilibrium. It was observed that the Nb_2_O_5_@H_2_TPP material exhibited 29% adsorption, whereas Nb_2_O_5_ reached 34%. The relatively high adsorption percentages observed for the methylene blue solution can be primarily attributed to the cationic nature of the dye combined with the anionic character of the photocatalyst surface at the solution’s native pH (∼pH 6.5). Under these conditions, the material exhibits a negative zeta potential Figure 9, which promotes adsorption via electrostatic attraction between opposite charges. The lower adsorption percentage for Nb_2_O_5_@H_2_TPP compared to Nb_2_O_5_ is attributed to the presence of porphyrin adsorbed onto the niobium pentoxide, which reduces dye adsorption by decreasing the number of available sites for Nb_2_O_5_–MB interaction.

### 2.4 Photocatalytic Assays

#### 2.4.1 Evaluation of the Effects of Time, MB Concentration, and Catalyst Mass

The methylene blue degradation reactions were initially conducted under white light (entry 1, Table 2), with a power of 3 W and in the absence of light (entry 2, Table 2). For these systems, a degradation percentage of methylene blue equal to or lower than 1% was observed, indicating the high stability of the dye both in the absence of light and under white light exposure. Control reactions conducted with only the dye in the presence of porphyrin (H_2_TPP) (entries 3 and 4, Table 2) did not show significant degradation percentages, which demonstrates that porphyrin alone, under visible light irradiation, does not exhibit photocatalytic activity—possibly due to the high aggregation of porphyrin in aqueous media, which reduces its luminescent properties.^53^ The Nb_2_O_5_ system showed no catalytic activity in the absence of light stimulation, whereas under light irradiation, a degradation percentage of 10% was observed. In this case, it is possible to propose that Nb_2_O_5_ possesses a suitable band gap, such that the energy of the incident photons enables the excitation of electrons from the valence band to the conduction band, generating electron–hole pairs. Species present in the medium with a reduction potential lower than that of the valence band of Nb_2_O_5_ can donate electrons to the valence band, whereas species with a reduction potential higher than that of the conduction band of Nb_2_O_5_ can accept excited electrons from the conduction band. In both cases, radical species are formed. Which act in the degradation of methylene blue.^54,55^

**Table 2:** Percentage of methylene blue Degradation in Systems Catalyzed by Nb_2_O_5_, H_2_TPP, and Nb_2_O_5_@H_2_TPP in the Absence of Light and under White Light Irradiation

entry^a^	light irradiation	Nb_2_O_5_ (mg)	H_2_TPP (mg)^b^	Nb_2_O_5_@H_2_TPP (mg)	degradation (%)
1	yes	–	–	–	1
2	no	–	–	–	0
3	yes	–	0.1	–	1
4	no	–	0.1	–	0
5	yes	10.0	–	–	10
6	no	10.0	–	–	0
7	yes	–	–	10.0	15
8	no	–	–	10.0	0

a The concentration of the methylene blue solution used was 5 × 10^–4^ mol L^–1^.

b The amount of H_2_TPP used was 0.1 mg, approximately 1000 times greater than that present in the material, due to the limitation of mass measurement.

The system employing MB and the Nb_2_O_5_@H_2_TPP material under white light irradiation (entry 7) exhibited an increase in degradation compared to the system using only Nb_2_O_5_ (entry 5, Table 2). It is proposed that upon white light irradiation, electrons in the porphyrins are excited from the HOMO to the LUMO, and during relaxation, electrons may be transferred from the porphyrin LUMO to the LUMO of Nb_2_O_5_. This process results in the formation of electron–hole pairs, which are responsible for generating radical species,^54^ capable of promoting dye degradation.

After this exploratory analysis, it was determined that the system employed for MB degradation could be optimized by identifying the significant variables and the optimal conditions for dye degradation. To achieve this, a statistical data analysis was performed based on a full factorial design of type 2^3^, with three replicates at the central point. The results obtained from the 2^3^ factorial design are presented in Table 3, and the corresponding statistical analysis results are illustrated in Figure 11.

**Table 3:** Percentage of methylene blue Degradation Photocatalyzed by Nb_2_O_5_@H_2_TPP

entry	MB (mol L^–1^)	time (h)	Nb_2_O_5_@H_2_TPP (mg)	Abs (*t*_0_)	Abs (*t*_*x*_)	degradation (%)
9	5 × 10^–4^	2.0	5.0	0.7515	0.7079	5.8
10	1 × 10^–3^	2.0	5.0	0.7877	0.7861	0.2
11	5 × 10^–4^	8.0	5.0	0.7558	0.6783	10.2
12	1 × 10^–3^	8.0	5.0	0.7877	0.7838	0.5
13	5 × 10^–4^	2.0	15.0	0.6372	0.472	25.9
14	1 × 10^–3^	2.0	15.0	0.6630	0.6350	4.2
15	5 × 10^–4^	8.0	15.0	0.6585	0.4436	32.6
16	1 × 10^–3^	8.0	15.0	0.6601	0.6373	3.5
17	7.5 × 10^–4^	5.0	10.0	0.7250	0.6997	3.5
18	7.5 × 10^–4^	5.0	10.0	0.7609	0.7068	7.1
19	7.5 × 10^–4^	5.0	10.0	0.6850	0.6695	2.3

**Figure 11:**
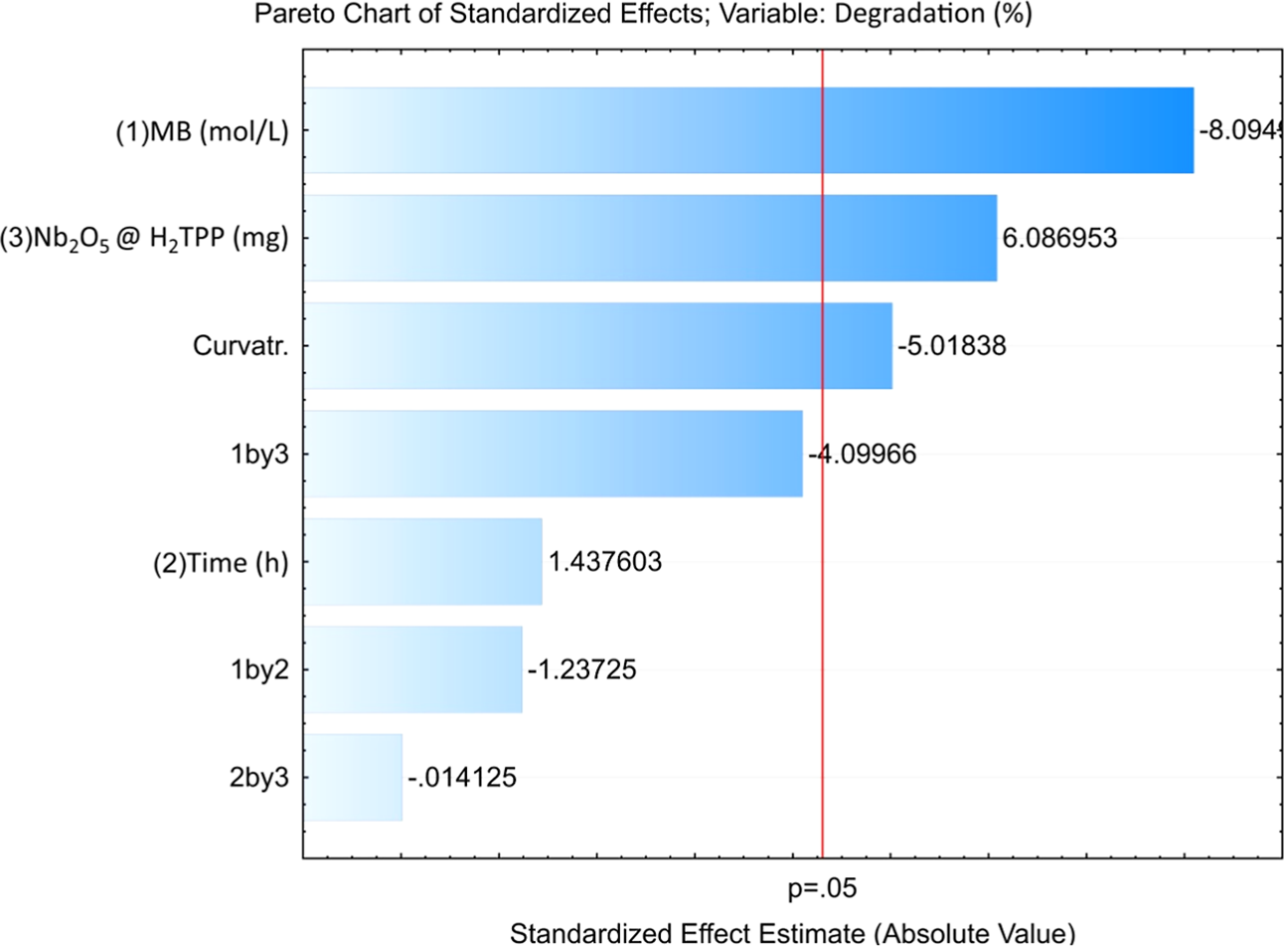
Pareto chart of the standardized effects from the full factorial 2^3^ design for the study of methylene blue degradation in systems catalyzed by Nb_2_O_5_@H_2_TPP under white light irradiation.

The statistical analysis identified a significant curvature in the response surface (Figure 11), which is more evident in the response surface plots shown in Figure 12. This reveals the limitations of the linear model and the need to adopt a nonlinear model. Additionally, a significant negative curvature suggests a response surface with downward concavity and the potential existence of a maximum point. Overall, the observed trends indicate that achieving higher degradation percentages would require reducing the dye concentration and increasing the catalyst mass. However, there is a limit to this, since decreasing the dye concentration and increasing the catalyst mass significantly enhance the adsorption percentage, which would preclude the analysis of the material’s photocatalytic activity.

**Figure 12:**
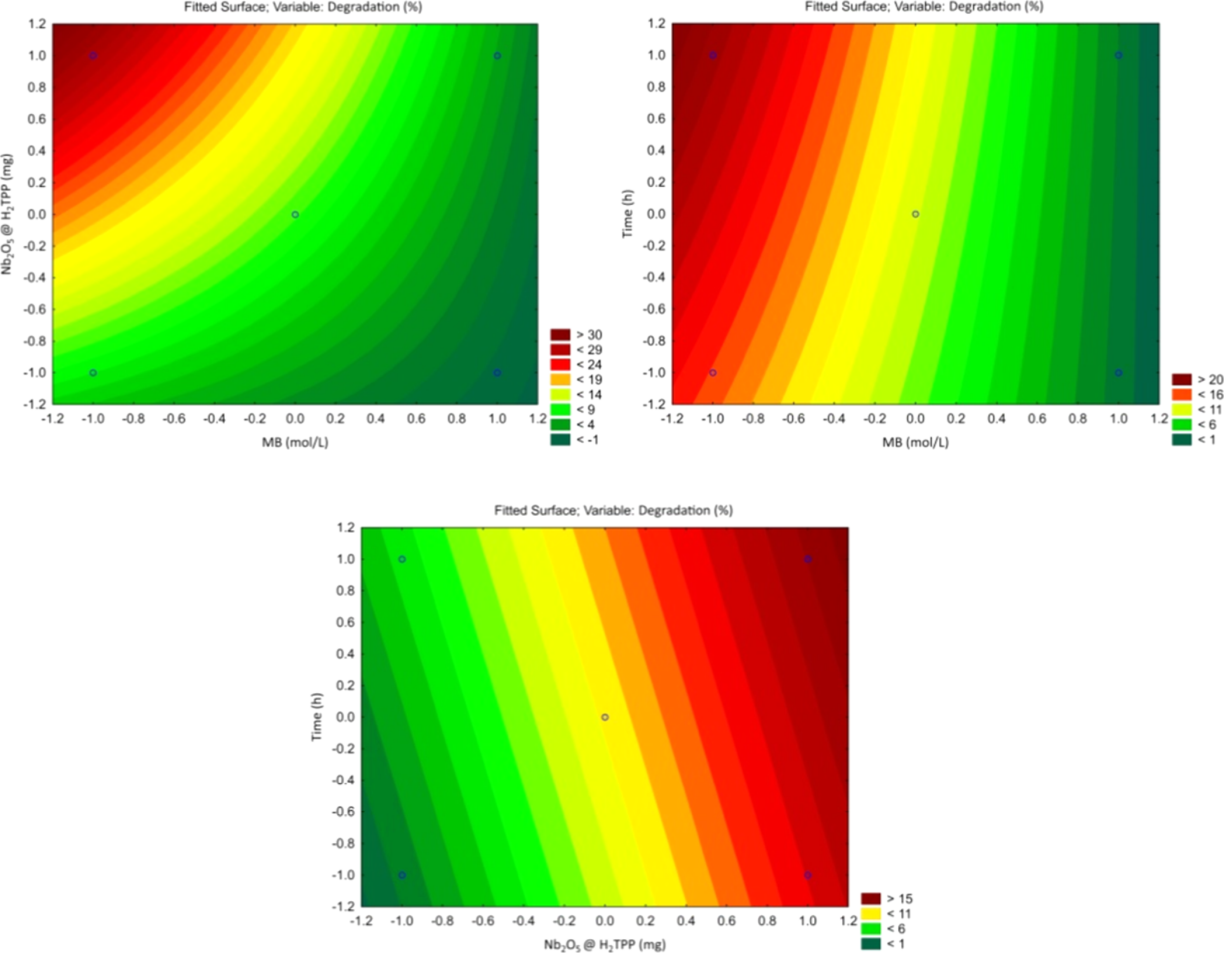
Response surfaces fitted from the full factorial 2^3^ design for the study of methylene blue degradation in systems catalyzed by Nb_2_O_5_@H_2_TPP under white light irradiation.

#### 2.4.2 Evaluation of Electromagnetic Radiation in MB Degradation Reactions

In the methylene blue degradation experiment using white light, which exhibits an emission spectrum across the entire visible region, a photocatalytic activity of 32% was observed when employing an MB concentration of 5 × 10^–4^ mol L^–1^ and 15 mg of the photocatalytic material Nb_2_O_5_@H_2_TPP. Consequently, the influence of different visible spectrum ranges (blue, green, and red) on methylene blue degradation was evaluated using the aforementioned dye concentration and catalyst mass (Table 4).

**Table 4:** Percentage of Degradation of 5 × 10^–4^ mol L^–1^ MB Photocatalyzed by Nb_2_O_5_@H_2_TPP under Blue, Red, and Green Light

entry	catalyst^a^	light	*t*_0_ ^b^	*t*_2h_ ^c^	*t*_4h_ ^d^	degradation (%)^e^
20	-	blue	0.8821	0.8871	0.8876	0
21	Nb_2_O_5_@H_2_TPP	blue	0.7083	0.4824	0.4696	34
22	-	green	0.8881	0.8870	0.8874	0.6
23	Nb_2_O_5_@H_2_TPP	green	0.7110	0.4271	0.4159	42
24	-	red	0.8875	0.8873	0.8831	0.5
25	Nb_2_O_5_@H_2_TPP	red	0.7176	0.4592	0.3946	44

a Catalyst mass used in these systems was 15 mg.

b MB absorbance (entries 20, 22, and 24). MB absorbance after 2 h of adsorption (entries 21, 23, and 25).

c MB solution absorbance after 2 h of exposure to electromagnetic radiation.

d MB solution absorbance after 4 h of exposure to electromagnetic radiation.

e The degradation percentage was calculated using eq 4, after 4 h of reaction.

The MB solution in the absence of the photocatalyst (Nb_2_O_5_@H_2_TPP) remained stable throughout the 4 h reaction under all studied light ranges (entries 20, 22, and 24). After irradiation with blue, green, or red light in systems containing the photocatalyst (entries 21, 23, and 25, Figure 13), a significant degradation percentage was observed within the first 2 h of reaction for all three systems studied, with no significant differences in degradation percentage during the subsequent 2 h.

**Figure 13:**
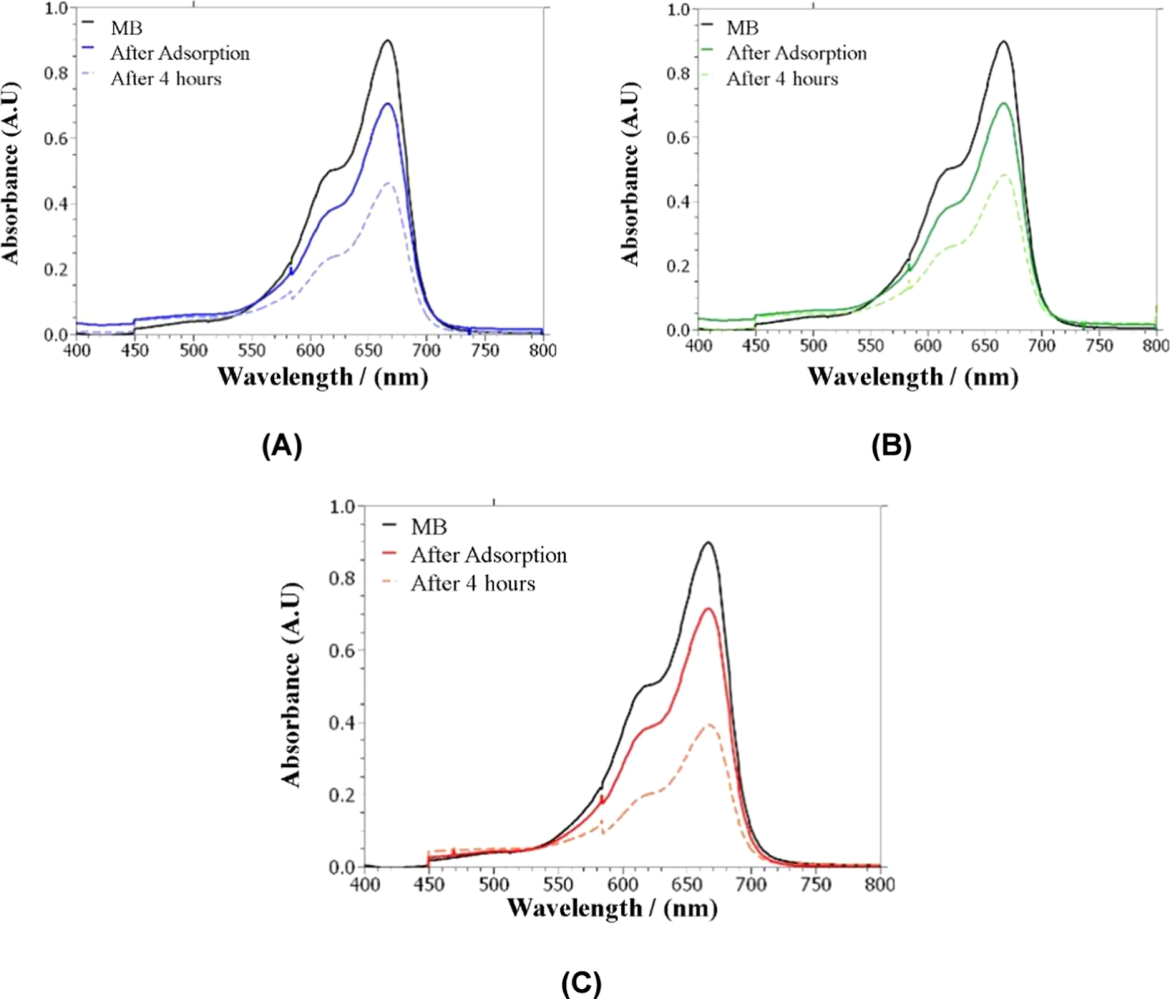
UV–vis absorption spectra of methylene blue degradation systems under light: (a) blue, (b) green, and (c) red.

The highest degradation percentages of MB were obtained when using green and red light, which may be related to the absorptions corresponding to the Q bands of the porphyrin in these regions. Although the porphyrin’s band with the highest molar absorptivity (Soret band) has a maximum at 421 nm (close to the blue region), the UV–vis spectrum of the blue light used spans 430–500 nm (Figure 3B), a region where there are no absorption maxima, which may reduce electronic excitation processes and explain the lower efficiency in methylene blue degradation when this radiation is employed. Additionally, the green and red LEDs exhibited higher irradiance, which can enhance the formation of radical species justifying the higher methylene blue degradation yields observed with the red and green LEDs compared to the blue LED, which had a lower illuminance value.

## 3 Conclusion

The developed photoreactor demonstrated stability throughout the 4 h reaction, exhibiting effective temperature control and consistent operation of the light source. Thus, it can be concluded that the constructed photoreactor has great potential for application in the degradation of emerging pollutants, in addition to presenting a low cost.

Based on XRD, UV-DRS, and SEM data, it can be proposed that the synthesis method employed was effective in obtaining the desired photocatalyst (Nb_2_O_5_@H_2_TPP) and that the incorporation of porphyrin into Nb_2_O_5_ did not significantly alter the surface morphology of this oxide, nor was there a significant change in the estimated band gap between Nb_2_O_5_ and the photocatalytic material Nb_2_O_5_@H_2_TPP.

In the methylene blue degradation reactions photocatalyzed by Nb_2_O_5_@H_2_TPP, a statistical study showed that the catalyst mass positively influences degradation, while the dye concentration has a negative effect. Furthermore, reaction time under the studied conditions did not significantly affect methylene blue degradation.

Overall, the obtained photocatalyst proved effective in the photodegradation of methylene blue solutions when exposed to blue, green, red, and white light, indicating the potential application of this low-cost material in real systems for pollutant degradation.

## 4 Methods

### 4.1 Materials and Methods

#### 4.1.1 Reagents

5,10,15,20-Tetraphenylporphyrin (H_2_TPP, Figure 1b) was purchased from Aldrich Chemical Co and was purified on an alumina column using CH_2_Cl_2_ as the solvent. The following reagents were used without a preliminary purification step: ammoniacal niobium oxalate (purchased from Companhia Brasileira de Metallurgia e Mineração (CBMM)), methylene blue (C_16_H_8_ClN_3_S·*x*H_2_O; Fluka), dichloromethane P.A. (CH_2_Cl_2_; Dinâmica), synthetic air (O_2_/N_2_; Linde). All other reagents and solvents were of analytical grade, without a preliminary purification step.

#### 4.1.2 Equipment

UV–vis spectra (190–1100 nm) were recorded on a Global Trade model GTA-97 spectrophotometer. UV–vis spectra with diffuse reflectance were performed on a Thermo Scientific Evolution 600 spectrometer (200–900 nm), using BaSO_4_ as reference. X-ray diffraction (XRD) analysis was carried out at room temperature (25 °C) on a Shimadzu XRD-6000, using Cu Kα radiation (λ = 1.5418 Å). The diffractograms were collected over a range of 10° to 80° at a scan rate of 2° min^–1^. The analysis of the specific surface area and average pore size of Nb_2_O_5_ was carried out on a Micromeritics ASAP 2020 at −195 °C, and the values were obtained using the Brunauer–Emmett–Teller (BET) and Barrett−Joyner−Halenda (BJH) methods, respectively. The sample was first pretreated for 3 h at 350 °C and under vacuum (2 μm of mercury). Scanning Electron Microscopy analysis was performed on a JEOL JSM-6610LV/TMP scanning electron microscope, with an X-ray spectrometer (XRS). The analyses were conducted under an accelerating voltage of 15 kV and low vacuum (1 mPa), with the analyzed samples being previously coated with gold. For FTIR characterization, KBr pellets were prepared using quantities of the synthesized solid. The pellets were prepared using pelletizers and a hydraulic press at 8 tons of pressure. Measurements were taken using a Shimadzu IR-Tracer 100 spectrometer, with a scan range of 400 to 4000 cm^–1^. A background measurement was performed using a KBr pellet without a sample. The TG analyses were performed using a Hitachi STA 7300 (Japan) instrument. Zeta potential analyses were carried out using a Zetasizer Nano Series instrument, model ZEN5600. The measurements were performed in aqueous solutions containing NaNO_3_ as the supporting electrolyte.

### 4.2 Photoreactor

The photoreactor was built using scrap materials and divided into two parts: the physical structure and the electrical circuit with automated temperature control.

#### 4.2.1 Physical Structure of the Photoreactor

The base chosen for the construction of the photoreactor was two cylindrical niches, the inside of which is 27 cm in diameter and 10 cm high, with hollow sides. This hollow part was designed to contain the electronic components, so that wires could be passed through to connect the electrical components without them being visible. The inside was completely covered in aluminum, which was fixed with gutter sealant. The outside was spray-painted matte black.

#### 4.2.2 Electrical Circuit—Temperature Control

For temperature control, a circuit was built using some scrap materials, such as 3 computer coolers and a DHT11 temperature sensor. An LCD display and an ATMega328—Arduino Nano microcontroller were purchased commercially. Arduino programming is described in the Supporting Information.

The three coolers were arranged around the bottom of the photoreactor. Two of these coolers were placed opposite each other (180° angle) and the third between them (90° from each cooler mentioned above).

#### 4.2.3 Electrical Circuit—Light Source

Regarding the light source, a 3W RGB LED bulb was chosen, which was attached to an E27 socket bulb, which was connected to a wire and a plug that was directly connected to the mains. The on/off switch and the choice of power and wavelength range were operated via an infrared remote control. To determine the emission spectrum of each of the colors (white, blue, green and red), a portable OCEAN OPTICS model USB4000-XR1-ES spectrophotometer with expanded UV-NIR range coupled to a fiber optic probe was used. The spectral irradiance measurements were performed using an OCEAN OPTICS fiber-optic spectrometer equipped with a 2048-pixel linear CCD detector, covering the spectral range from 350 to 1050 nm. The system was calibrated with an Ocean Optics DH-3-Plus light source. In addition, a lux meter was developed, based on a BH1750FVI sensor associated with the Arduino Nano (detailed description in the Supporting Information) to determine the illuminance associated with each color.

### 4.3 Photocatalyst

#### 4.3.1 Synthesis of Nb_2_O_5_

Nb_2_O_5_ was obtained from the calcination of ammoniacal niobium oxalate NH_4_[NbO­(C_2_O_4_)_2_(H_2_O)_2_]·3H_2_O, which was carried out by heating the oxalate to a temperature of 550 °C for 4 h, at a heating rate of 10 °C/min, under a flow of 50 mL min^–1^ of synthetic air. The choice of calcination temperature was based on data from thermogravimetric analysis carried out on NH_4_[NbO­(C_2_O_4_)_2_(H_2_O)_2_]·3H_2_O, under a flow of synthetic air, in the temperature range from 25 to 1000 °C. The calcined solid was passed through an 80-mesh sieve to homogenize the particle size of the synthesized Nb_2_O_5_.

#### 4.3.2 Synthesis of Nb_2_O_5_@H_2_TPP

Twenty mL of a solution of H_2_TPP in CH_2_Cl_2_ with a concentration of 2.5 × 10^–4^ mol L^–1^ was added to a 50 mL round-bottom flask, followed by 500 mg of Nb_2_O_5_, in a ratio of 1 × 10^–5^ mol of porphyrin for every 1 g of Nb_2_O_5_. The system was kept at 35 °C under magnetic stirring for 2 h. After this time, another 5 mL of CH_2_Cl_2_ was added. The system was kept under magnetic stirring and heating at 35 °C until the solvent had completely evaporated. The crude material was dried in an oven for 24 h at 80 °C and then stored in a desiccator.

#### 4.3.3 Adsorption Tests

In a 10 mL penicillin flask, 10 mg of the photocatalytic material, Nb_2_O_5_@H_2_TPP (1 × 10^–7^ mol of H_2_TPP) and 4.0 mL of an aqueous methylene blue solution of concentration 1 × 10^–3^ mol L^–1^ were added. This system remained under magnetic stirring, in the absence of light stimulation and was monitored by absorption spectroscopy in the UV–vis region every 30 min for 180 min. Five minutes before the analysis, the magnetic stirring was stopped and after this time an aliquot of 30 μL of the solution was added to a cuvette containing 2.0 mL of distilled water, and then the spectrum was recorded.

#### 4.3.4 Photocatalytic Tests

The methylene blue photodegradation reactions were carried out on a microscale, in 10 mL penicillin flasks, with thermal control (25 ± 2 °C), under atmospheric pressure, using constant oxygenation by bubbling air with a Boyu model S510 air compressor, 4 L min^–1^, using distilled water as the solvent. The reactions were carried out following a complete factorial experimental design of type 2^3^ with three replicates at the central point. The following variables were studied: reaction time, concentration of the methylene blue solution and the mass of the photocatalyst. The photocatalytic material (Nb_2_O_5_@H_2_TPP) and 4.0 mL of an aqueous solution of methylene blue were added to the penicillin vial. This system remained under magnetic stirring for 2 h in the dark so that the adsorption/desorption equilibrium was established. After this time, the system was exposed to electromagnetic radiation, using blue, green, red and white light.

The percentage of degradation of the dye was determined by absorption spectroscopy in the UV–vis region, using eq 4. The analyses were carried out in the same way as in the study to determine the percentage of adsorption.

4 $$\%\;Bleaching=100\times \frac{Abs\left(0\right)-Abs\left(t\right)}{Abs\left(0\right)}$$

The absorbance at time zero (Abs(0)) refers to the absorbance after the adsorption process and the absorbance at time *t* (Abs­(*t*)) refers to the absorbance at the stipulated time. All the tests were carried out at least in duplicate and the results expressed are the average of the values obtained. Control reactions were carried out in the same conditions described for the photocatalyzed reactions, except: (1) in the absence of any photocatalyst; (2) with porphyrin-free Nb_2_O_5̈_in place of Nb_2_O_5_@H_2_TPP; (3) with H_2_TPP dispersed in water place of Nb_2_O_5_@H_2_TPP; and (4) in the dark.

#### 4.3.5 Statistics

In order to evaluate the effect of studied variable (MB (mol L^–1^), time (h), Nb_2_O_5_@H_2_TPP (mg)) on photocatalytic degradation a multivariate factorial experimental design was carried out.^56^ Statistical calculations for the 2^3^ full factorial design with central point to obtain the standardized effect estimates, as well as ANOVA significance evaluation by Pareto chart of the effect estimates using pure error term, were carried out by Statistica 12.5 package (Statsoft) at 95% confidence level.

## Supplementary Material



## Notes

Arduino programming language; Arduino-based script for real-time light intensity measurement; detailed cost analysis of the photoreactor; budget estimates for commercial photoreactors; complementary analyses; and additional technical details (PDF)
