# Supplementary material for: Development of a Low-Cost Microphotoreactor from Recycled Materials: Application to Nb2O5@H2TPP-Catalyzed Methylene Blue Degradation
Source: ACS Omega. 2026 Jan 22;11(4):5421–35. doi: 10.1021/acsomega.5c08779 (PMC12878737; doi:10.1021/acsomega.5c08779)
Supplement: Supplementary file 1 [file ao5c08779_si_001.pdf]

SUPPORTING INFORMATION

Development of a low-cost micro-photoreactor from recycled materials: application to Nb_2O_5@H_2TPP-catalyzed methylene blue degradation

Lívia Silva de Andrade[1], João Victor Docílio Pereira[1], Tiago Souza Brasil[1], Clarissa B. da. S. Neves[2], Felipe Breno Campos Marinho[3], Júlio Santos Rebouças[3], Sivanildo da Silva Borges[1], Fábio Santos de Oliveira[4], Clarivaldo Santos Souza[1], Gilson DeFreitas-Silva[5], Denilson Santos Costa*[2], Vinicius Santos da Silva[1*].

*e-mail: vinicius.sdasilva@ufrb.edu.br and denilsoncosta@ufba.br

[1] Centro de Ciências Exatas e Tecnológicas – Universidade Federal do Recôncavo da Bahia, 44380-000. Cruz das Almas, BA, Brazil.

[2] Instituto de Química, Universidade Federal da Bahia, 40170-115. Salvador, BA, Brazil.

[3] Departamento de Química, Centro de Ciências Exatas e da Natureza, Paraiba Federal University, *58051-900, João Pessoa, PB,* Brazil.

[4] Centro de Ciências da Saúde, Universidade Federal do Recôncavo da Bahia, 44570-000. Santo Antônio de Jesus, BA, Brazil.

[5] Departamento de Química, Instituto de Ciências Exatas, Universidade Federal de Minas Gerais, 31270-901. Belo Horizonte, MG, Brazil.

1 - Arduino Programming Language

The programming language used in the photoreactor for temperature control is described below.

```
#include <DHT11.h>
#include <LiquidCrystal_I2C.h>
#include <Wire.h>


DHT11 dht11(A0);

int c1 = 6;
int c2 = 3;

void display_it();
void ler_temp();

LiquidCrystal_I2C lcd(0x27,16,2);

void setup() {
  // put your setup code here, to run once:
  pinMode(c1, OUTPUT);
  pinMode(c2, OUTPUT);
  lcd.init();
  lcd.setBacklight(180);
  Serial.begin(9600);
}

void loop() {
  it();
}

void display_it(){
  float temperature = dht11.readTemperature();
  lcd.setCursor(2,0);
  lcd.print("Temperatura:");
  lcd.setCursor(5,1);
  lcd.print(temperature, 1);
  lcd.setCursor(9,1);
  lcd.print((char)223); // °
  lcd.print("C");
  Serial.println(temperature);
  delay(50);
}

void ler_temp(){
  float temperature = dht11.readTemperature();
  if (temperature != DHT11::ERROR_CHECKSUM && temperature !=
DHT11::ERROR_TIMEOUT)
    {
      Serial.print("Temperature: ");
      Serial.print(temperature, 1);
      Serial.println(" °C");
      lcd.setCursor(2,0);
      lcd.print("Temperatura:");
```

```
      lcd.setCursor(6,1);
      lcd.print(temperature, 1);
      lcd.setCursor(8,1);
      lcd.print((char)223); // °
      lcd.print("C");
    }
    else
    {
      Serial.println(DHT11::getErrorString(temperature));
    }
    delay(1000);
}

void it(){
  float temperature = dht11.readTemperature();
  if (temperature != DHT11::ERROR_CHECKSUM && temperature !=
DHT11::ERROR_TIMEOUT){
    display_it();
    if(temperature >= 26){
      digitalWrite(c1, LOW);
      digitalWrite(c2, LOW);
    }
    else {
      digitalWrite(c1, HIGH);
      digitalWrite(c2, HIGH);
    }
  }
  else{
    Serial.println(DHT11::getErrorString(temperature));
    lcd.clear();
    lcd.setCursor(5,0);
    lcd.print("Erro,");
    lcd.setCursor(2,1);
    lcd.print("Sensor DHT 11");
    digitalWrite(c1, LOW);
    digitalWrite(c2, LOW);
  }
}
```

2 – Arduino script for real-time measurement of luminous intensity (lux) using the BH1750 light sensor

2.1 Required Materials

Arduino Nano, 1 BH1750FVI light sensor and Jumper wires.

2.2 Wiring Scheme

- **VCC** of the BH1750FVI → to **5V** pin on the Arduino Nano
- **GND** of the BH1750FVI → to **GND** pin on the Arduino Nano
- **SDA** of the BH1750FVI → to **A4** pin on the Arduino Nano (SDA)
- **SCL** of the BH1750FVI → to **A5** pin on the Arduino Nano (SCL)

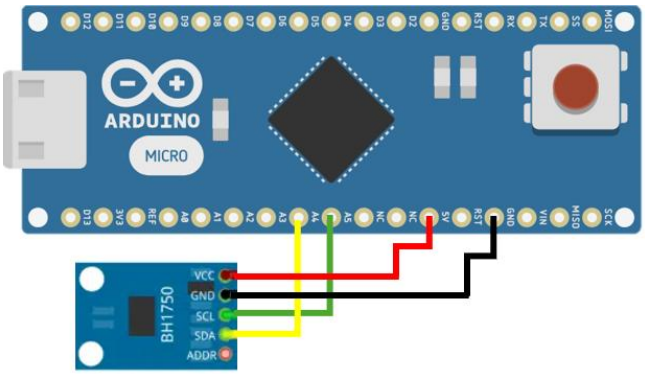

Figure S1 – Schematic representation of the connections between the Arduino Nano and the BH1750 light sensor.

This script was used to monitor light intensity (in lux) inside the photoreactor during photocatalytic experiments. It utilizes an Arduino Nano microcontroller, the BH1750 ambient light sensor, and the I2C communication protocol. The code initializes the sensor, reads the illuminance values at 1-second intervals, and outputs the data to the serial monitor.

Programming Language

#include <BH1750.h> // Includes the BH1750 library, which enables communication with the BH1750 light sensor.

#include <Wire.h> // Includes the Wire library, which enables I2C communication.

BH1750 lightMeter; // Creates an instance of the lightMeter object from the BH1750 class.

void setup() {

  Serial.begin(9600); // Initializes serial communication at a rate of 9600 bps.

  // Initializes the I2C bus. The BH1750 library does not do this automatically.

  Wire.begin();

  // For ESP8266, you can select the SCL and SDA pins using Wire.begin(D4, D3);

  // For Wemos / Lolin D1 Mini Pro and the ambient light shield, use

  // Wire.begin(D2, D1);

  lightMeter.begin(); // Initializes the BH1750 sensor.

  delay(100); // Waits 100 milliseconds to ensure the sensor is ready.

```
  Serial.println(F("BH1750 Test begin")); // Sends a message to the serial monitor indicating the test has started.
}

void loop() {
  float lux = lightMeter.readLightLevel(); // Reads the light level in lux from the BH1750 sensor and stores it in the variable lux.
  Serial.print("Light: "); // Prints the string "Light: " to the serial monitor.
  Serial.print(lux); // Prints the value of lux read.
  Serial.println(" lx"); // Prints " lx" to indicate the unit of measurement (lux).
  delay (1000); // Waits 1 second before repeating the loop.
}
```

2.3 Results

Using the custom-built luxmeter, 10 measurements were performed at 1-second intervals for the LED lamp inside the photoreactor, assessing light emissions in white, green, red, and blue modes (Table S1).

Table S1 - Relationship between illuminance, and colors.

Color	M1 (lx)	M2 (lx)	M3 (lx)	M4 (lx)	M5 (lx)	M6 (lx)	M7 (lx)	M8 (lx)	M9 (lx)	M10 (lx)
**White**	704.33	704.00	705.67	705.83	705.83	705.67	704.22	705.00	705.00	705.00
**Blue**	71.67	72.50	72.00	71.33	71.67	72.5	71.67	71.67	72.33	72.66
**Green**	439.17	439.17	439.00	437.33	440.00	440.00	440.00	441.17	440.00	441.33
**Red**	249.17	248.33	250.00	250.00	250.83	250.83	251.67	252.50	253.33	254.17

The luminous flux was determined using Equation S1.

$$\boldsymbol{F}(\boldsymbol{lm}) = \boldsymbol{I}(\boldsymbol{lx}) * \boldsymbol{A}\left(\boldsymbol{m^2}\right) \qquad \text{Equation S1}$$

Where:

- *F* is the luminous flux, in lumens (lm);
- *I* is the illuminance, in lux (lx);
- *A* is the illuminated surface area, in square meters (m²).

2.4 Costs associated with the construction of the photoreactor.

Table S2: Relationship between the materials required for the construction of the photoreactor and their respective prices.

	Material	Amount	Parcial Price in Brazil[1]		Total Price in Brazil[1]	
Electrical Structure	Arduino Nano	1	R$	25.09	R$	25.09
	Jumper Wires Kit	1	R$	14.30	R$	14.30
	Relays	3	R$	11.95	R$	35.85
	I2C LCD Display	1	R$	25.00	R$	25.00
	Power Source	2	Recycled		-	
	Coolers	3	Recycled		-	
	PCB board	1	R$	2.15	R$	2.15
	Tin	1	R$	6.96	R$	6.96
	RGB Light	1	R$	59.17	R$	59.17
	DHT11 Sensor	1	R$	7.50	R$	7.50
Physical Structure	Hot Glue	10	R$	1.25	R$	12.50
	Mettalic Ribbon	1	R$	19.00	R$	19.00
	Spray Painting	1	R$	14.90	R$	14.90
	Wooden niche	2	Recycled		-	
	Screw	1	Recycled		-	
	Zinc Plate	1	Recycled		-	
	Total Cost in Brazil				R$	222.42

1 – The quotations were obtained in October 2025, during which the exchange rate was US$ 1.00 = R$ 5.36. Total cost in US$ 41.50.

Table S3 – Commercial Photoreactors: Suppliers, Specifications, and Pricing

**Supplier**	**Model**	**Price**	**Site***
HepatoChem / EvoluChem	PhotoRedOx Box UV-Vis (HCK1022-01-001)	**US$5,250.00**	https://hepatochem.com/photoreactor-order/ . (HepatoChem)
Luzchem Research	LZC series (ex.: LZC-4 / LZC-5b)	**US$15,943.50**	https://luzchem.com/products/luzchem-lzc-photoreactor
Sigma-Aldrich	Penn PhD Photoreactor M2	**US$8,960.00**	https://www.sigmaaldrich.com/BR/pt/product/sial/z744035
Sigma-Aldrich	SynLED Parallel Photoreactor 2.0	**US$ 3,240.00**	https://www.sigmaaldrich.com/BR/pt/product/aldrich/z744080

* Survey conducted in December 2025.

2.5 Photocatalyst

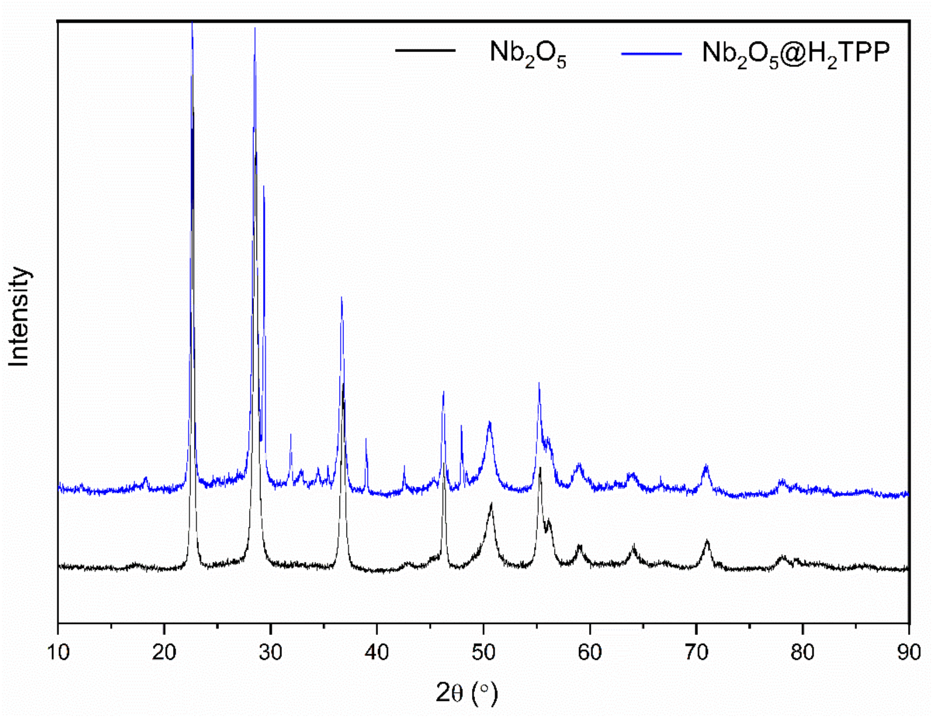

Figure S2 - Diffractogram of Nb_2O_5@H_2TPP.

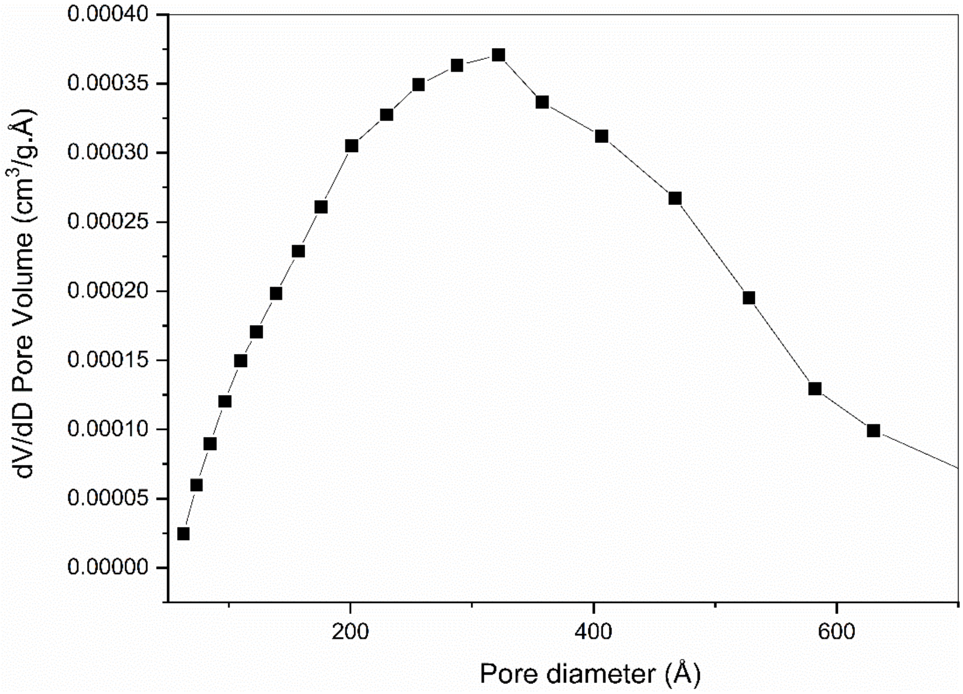

Figure S3 - Average pore size of Nb_2O_5.

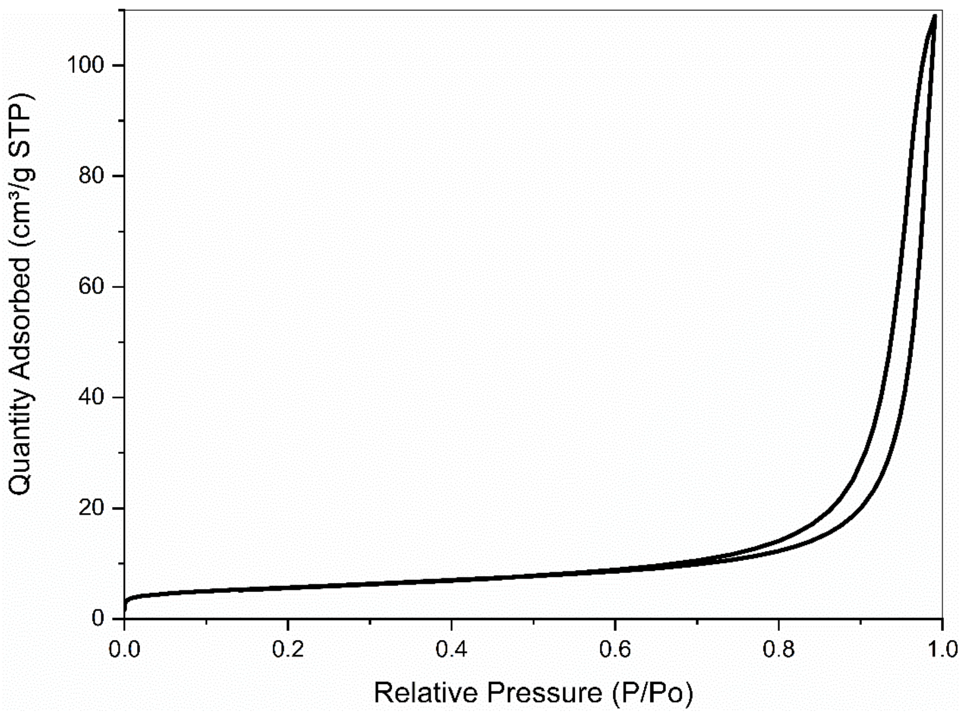

Figure S4 - N_2 adsorption-desorption isotherm of Nb_2O_5.
